# A gut microbiome tactile teaching tool and guided-inquiry activity promotes student learning

**DOI:** 10.3389/fmicb.2022.966289

**Published:** 2022-12-22

**Authors:** Parker T. Shoaf, Katie S. French, Noah J. Clifford, Erin A. McKenney, Laura E. Ott

**Affiliations:** ^1^Department of Biology, University of North Carolina at Chapel Hill, Chapel Hill, NC, United States; ^2^Department of Forestry and Environmental Resources, North Carolina State University, Raleigh, NC, United States; ^3^Department of Applied Ecology, North Carolina State University, Raleigh, NC, United States; ^4^Carolina Biology Education Research Laboratory, University of North Carolina at Chapel Hill, Chapel Hill, NC, United States

**Keywords:** gut microbiome, short chain fatty acids, tactile teaching tools, guided-inquiry learning, undergraduate learning, remote learning, inclusive learning, active learning

## Abstract

The gut microbiome and its physiological impacts on human and animal health is an area of research emphasis. Microbes themselves are invisible and may therefore be abstract and challenging to understand. It is therefore important to infuse this topic into undergraduate curricula, including Anatomy and Physiology courses, ideally through an active learning approach. To accomplish this, we developed a novel tactile teaching tool with guided-inquiry (TTT-GI) activity where students explored how the gut microbiome ferments carbohydrates to produce short chain fatty acids (SCFAs). This activity was implemented in two sections of a large-enrollment Human Anatomy and Physiology course at a research intensive (R1) university in the Spring of 2022 that was taught using a hyflex format. Students who attended class in person used commonly available building toys to assemble representative carbohydrates of varying structural complexity, whereas students who attended class virtually made these carbohydrate structures using a digital learning tool. Students then predicted how microbes within the gut would ferment different carbohydrates into SCFAs, as well as the physiological implications of the SCFAs. We assessed this activity to address three research questions, with 182 students comprising our sample. First, we evaluated if the activity learning objectives were achieved through implementation of a pre-and post-assessment schema. Our results revealed that all three learning objectives of this activity were attained. Next, we evaluated if the format in which this TTT-GI activity was implemented impacted student learning. While we found minimal and nonsignificant differences in student learning between those who attended in-person and those who attended remotely, we did find significant differences between the two course sections, which differed in length and spacing of the activity. Finally, we evaluated if this TTT-GI approach was impactful for diverse students. We observed modest and nonsignificant positive learning gains for some populations of students traditionally underrepresented in STEM (first-generation students and students with one or more disabilities). That said, we found that the greatest learning gains associated with this TTT-GI activity were observed in students who had taken previous upper-level biology coursework.

## 1. Introduction

It is well established that active learning pedagogies promote student achievement in science, technology, engineering, and mathematics (STEM) courses (Haak et al., 2011; Freeman et al., 2014). In fact, active learning approaches have been particularly impactful for diverse students, to include persons excluded because of their ethnicity or race (PEERs; Beichner et al., 2007; Asai, 2020; Gordy et al., 2020; Kressler and Kressler, 2020; Theobald et al., 2020). While numerous active learning modules have been developed within the field of biology, more are needed to promote the success of diverse student populations. This is especially important for emerging areas within the field of biology, such as the gut microbiome and its impact on human and veterinary health and disease (Turnbaugh et al., 2007; Pilla and Suchodolski, 2020). In fact, the human microbiome has been described as a distinct organ system (Baquero and Nombela, 2012), which necessitates inclusion of this important topic within human and comparative physiology-focused courses.

Numerous active learning modules have been developed for students to explore microbial communities. These include inquiry-based activities, where students formulate hypotheses and either conduct experiments or analyze existing data sets to form conclusions (Wang et al., 2015; Freeman et al., 2016; Lentz et al., 2017; Genné-Bacon and Bascom-Slack, 2018; Goller and Ott, 2020; Sewall et al., 2020); simulations and/or modeling activities (Estes, 2015; Robertson-Albertyn et al., 2016; Rabelo-Fernandez and Rios-Velazquez, 2021); and gamification activities (Coil et al., 2017). Some of these activities provide novice learners the opportunity to explore the physiological connection between the gut microbiome and its host. For example, Estes (2015) describes an activity where students in an undergraduate microbiology course explore the community of microbes that inhabit the human digestive system and how disturbance events, such as antibiotics, can impact these communities. Alternatively, a classroom based undergraduate research experience (CURE) was developed where students performed laboratory techniques to explore how dietary modifications, such as increasing dietary fiber, impacted the microbiome of the students enrolled in the class (Sewall et al., 2020). Another increasingly popular active learning approach, especially in research-based courses, is for students to collect biological data to pool into larger, sometimes nation-wide, databases (Freeman et al., 2016; Genné-Bacon and Bascom-Slack, 2018). For example, Freeman et al. (2016) detailed a project where students collected data on their own facial microbiomes, along with demographic and lifestyle variables, to address novel questions, such as how one’s diet impacts their microbial diversity. While these active learning modules provide an effective mechanism to teach the physiological relevance of the microbiome, many are laboratory-based activities (Freeman et al., 2016; Genné-Bacon and Bascom-Slack, 2018; Goller and Ott, 2020; Sewall et al., 2020) that are not feasible for a non-laboratory course. Additionally, many of these published activities also focus on novice learners and may not be appropriate for an upper-level undergraduate Human Anatomy and Physiology course (Estes, 2015; Robertson-Albertyn et al., 2016; Coil et al., 2017; Rabelo-Fernandez and Rios-Velazquez, 2021). Further, additional considerations must be made for large-enrollment courses that are offered in a hyflex format, where students can attend class either in-person or *via* a virtual live stream option (e.g., Zoom).

Numerous active learning approaches can be used to teach complex biological topics, such as the physiological impacts of the gut microbiome. It is often difficult, however, to teach these concepts in a way that allows students to visualize complex biological processes. Ramirez and Gordy (2020) describe several approaches to enable student visualization of biological processes, including instructor-led demonstrations, student-driven three-dimensional (3D) printing projects, structure-focused in-class activities, interactive classroom activities, and tactile teaching tools with guided inquiry (TTT-GI).

The TTT-GI approach blends both the use of tactile teaching tools (TTT) and guided inquiry (GI) learning. The use of TTT enhances student learning through manipulation of 3D models (Cooper and Oliver-Hoyo, 2017; Howell et al., 2018; Newman et al., 2018; Gordy et al., 2020; Ramirez and Gordy, 2020). Development of TTT typically involves either commonly available objects, such as craft supplies (DeBruyn, 2012; Mayorga et al., 2012; Gehret, 2017), or the use 3D printed objects (Howell et al., 2019; Kerwin, 2019; Gordy et al., 2020). Further, TTT are designed to be accessible and inclusive of a wide range of individuals following Universal Design for Learning (UDL) principles, which ensure that lessons are flexible and adaptable to accommodate a range of learning needs (Rose and Meyer, 2006; Burgstahler and Corey, 2008; Rappolt-Schlichtmann et al., 2012; Meyer et al., 2014; Hasper et al., 2015). For example, a 3D printed *lac* operon TTT was designed to respond with vibration when the RNA polymerase bound to the -10/-35 regions of the *lac* promoter (Gordy et al., 2020). This provides an inclusive learning experience for all students, including those who with disabilities such as visual impairment (Hasper et al., 2015).

TTT-GI activities also draw on constructivist pedagogies (Piaget, 1950; Bodner, 1986; Bodner et al., 2001; Eberlein et al., 2008) and specifically emphasize that instead of transferring knowledge from instructor to students in a traditional didactic environment, students must actively draw on their previous learning to build new knowledge. This requires students to work through the learning cycle, where they first explore the topic by drawing on previous knowledge; then engage in concept invention, where they explore a specific concept in detail; and then finally apply their new knowledge to a new scenario (Abraham, 2005; Eberlein et al., 2008; Cracolice, 2009). Most constructivist pedagogies, including process oriented, guided inquiry learning (POGIL), are examples of cooperative learning, where students work in small groups to complete a structured activity *via* student-to-student interactions that involve positive interdependence as well as individual accountability (Johnson et al., 1991, 2010). In a POGIL classroom, students work in teams to complete guided learning activities that have been specifically designed to walk them through the learning cycle (Moog et al., 2006, 2009; Eberlein et al., 2008). Large-enrollment courses have particularly benefitted from cooperative learning pedagogies (Cooper, 1995) and guided-learning approaches have been shown to be an effective way to teach topics within life science disciplines (Loertscher and Minderhout, 2011; Trout, 2012; Gordy et al., 2020), including Anatomy and Physiology courses (Brown, 2010; Jensen, 2014).

**Figure 1:**
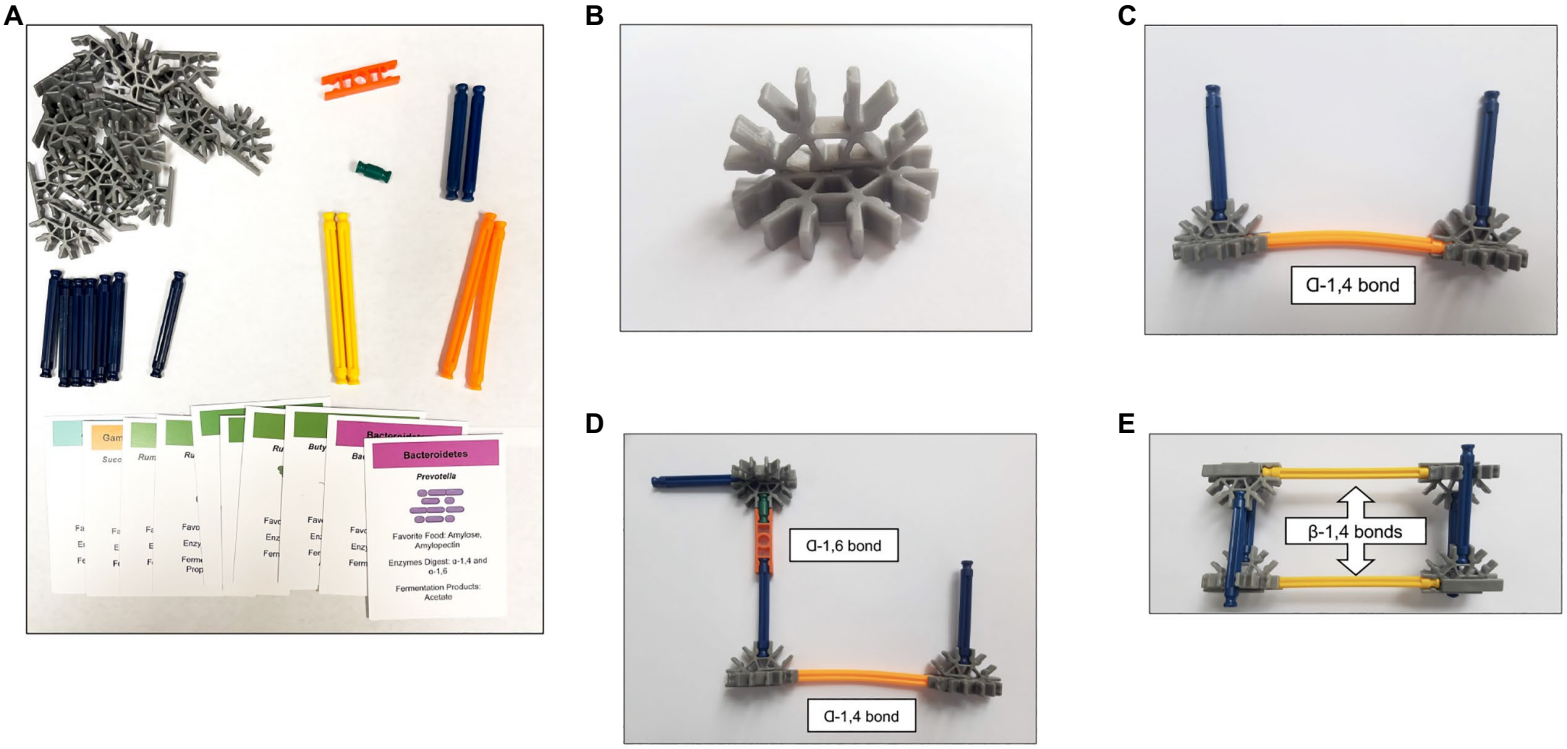
Components of the TTT-GI kit. **(A)** The tactile teaching tool aspect of the kit includes 33 specific K’nex pieces and a deck of 9 double-sided cards. **(B)** K’nex representation of a single glucose molecule. **(C)** K’nex representation of amylose. **(D)** K’nex representation of amylopectin. **(E)** K’nex representation of cellulose.

We have developed a TTT-GI activity that focuses on the gut microbiome and the physiological impacts of specific diets, which we implemented in a hyflex, large-enrollment Human Anatomy and Physiology course at an R1 institution. We used a Backward Design approach (Wiggins and McTighe, 2005) to design the activity, where we first established student learning objectives or SLOs (Table 1) that were mapped to revised Bloom’s Taxonomy of Educational Objectives (Anderson and Krathwohl, 2001). We then developed summative assessment questions to evaluate students’ mastery of the activity SLOs (Table 1). Finally, we developed our TTT-GI activity, which consisted of three phases: pre-lesson work, in-class part 1, and in-class part 2 (Figure 1). The activity was purposely developed for a hyflex learning environment, with activity adaptations for both in-person and remote learners. We completed a robust assessment of this activity that specifically addressed three research questions. Our first research question was to determine if the activity promoted attainment of the activity SLOs (Table 1). The second research question evaluated if there were differences in the attainment of the activity learning objectives based on how students attended class (e.g., remote or in-person). The final research question evaluated if we observed differences in attainment of the activity SLOs among different student populations. Herein we describe our TTT-GI activity and assessment results that specifically address our three research questions. We also discuss limitations of this activity, as well as possible modifications.

**Table 1:** Gut microbiome TTT-GI learning objectives mapped to revised Bloom’s Taxonomy and associated assessment questions.

SLO	Learning objectives	Bloom’s taxonomy level	Assessment question(s)
SLO-1	Compare the role of different bacteria in the digestion of different carbohydrates	Analyze	1, 5, 8
SLO-2	Explain the process by which bacteria ferment carbohydrates to produce short chain fatty acids (SCFA)	Understand	4
SLO-3	Predict the consequences of different diets and bacteria in the digestive system for overall health	Evaluate	2, 3, 5, 6, 7, 9

## 2. Materials and methods

This study was granted exempt status by the University of North Carolina at Chapel Hill Institutional Review Board (IRB). All students were required to participate in the activity as a course requirement, but only students who consented to the use of their educational data were included in analyses.

### 2.1. Description of the course

This activity was implemented in two sections of a high-enrollment, 200-level Human Anatomy and Physiology course at a public, R1 institution during the Spring of 2022. One section, called the 75-min section, met for 75 min twice a week and had a total of 169 enrolled students. The other section, called the 50 min section, met for 50 min three times per week and had a total of 183 students enrolled. Both sections were taught in the same stadium-seating lecture hall classroom by the same PhD-level instructor, with the equivalent content covered in both sections. Each section also had a team of nine undergraduate peer instructors, who attended class and helped facilitate in-class learning. These peer instructors also held weekly virtual review sessions on course topics. The pre-requisite for the course was a 100-level Introductory Biology course. Students could have earned credit for this Introductory Biology course through Advanced Placement (AP) credit, transfer credit, a placement exam, or by enrolling in the course at the institution. A comparison of the characteristics and student populations for each section is detailed in Supplementary Table 1.

The course was taught in a high-structure format that has been previously shown to benefit biology majors, particularly in large-enrollment courses (Haak et al., 2011; Eddy and Hogan, 2014). Before class, students completed an assigned reading from the required textbook or other source along with optional but encouraged guided reading questions (GRQs), before completing an online homework assignment. The in-class component was a mixture of lecture and active learning, where students completed practice problems or activities and engaged in small group discussions related to the course content. Due to the ongoing COVID-19 pandemic, each section of the course was taught in a hyflex format, meaning that throughout the semester, students had the option of attending class either in-person or *via* a synchronous virtual live stream as needed. The instructor always attended class in-person and had a team of undergraduate peer instructors facilitate the virtual live stream option. Post-lesson homework was assigned at regular intervals (every 3–4 lessons) and consisted of higher order multiple choice questions (8 total post-lesson homework assignments per semester). Over the course of the 15-week semester there were 24 lessons covering all 11 organ systems plus the microbiome TTT-GI described here. These lessons were divided into four units, with each unit culminating in a unit exam. Our TTT-GI lesson was in unit 4 and presented immediately after a lesson focused on the digestive system.

### 2.2. Development of the TTT-GI activity

For students participating in the activity in-person, the TTT-GI kit was designed to include all necessary K’nex pieces to build three separate carbohydrate structures representing amylose, amylopectin, and cellulose (Figure 1). The kit was designed such that two interlocked gray half-circle K’nex pieces represent a basic 6-carbon glucose unit or monomer (Figure 1B). Specific K’nex rod pieces were used to represent different glycosidic bonds (α-1,4, α-1,6, and β-1,4) in the three carbohydrate structures (Figures 1C–E). The kit also included a card deck consisting of 9 double-sided cards. The front of each card included information about a specific microorganism found in the gut. The back of each card outlined the specific carbohydrates each microbe digests and the SCFA fermentation products each microbe produces, with a picture of a K’nex piece depicted to represent the carbons in each of the SCFA. The specific K’nex pieces and card deck template needed for each kit can be found on the kit assembly document available at: https://stembuild.ncsu.edu/lesson-plan/bacterial-fermentation/. We found that a standard micropipette tip box worked well to house and distribute each individual kit to students, with one kit needed per group of 3–4 students. Along with the kit, each student team needed access to the kit instructions (available at: https://stembuild.ncsu.edu/lesson-plan/bacterial-fermentation/). The instructions were developed to provide step-by-step illustrated guides to construct the three carbohydrate structures.

For students participating remotely, the activity was modified to allow students to engage in an online environment. A digital playground Word document was generated that contained digital versions of each K’nex piece included in the kit described above. The images on the digital playground document were freely moveable, allowing students to manipulate the images to build the three carbohydrate structures on the following pages. A remote card deck, which contained all nine cards described above, was made with the cards presorted into three unique hands for the students to use as they progressed through the activity. All documents needed to implement this activity in a remote setting are available at: https://stembuild.ncsu.edu/lesson-plan/bacterial-fermentation/.

Both remote and in-person students had access to the same guided inquiry worksheet, which was made available electronically *via* the course Learning Management System (LMS). This document is available at: https://stembuild.ncsu.edu/lesson-plan/bacterial-fermentation/. Part 1 of the guided inquiry activity consisted of three sections with accompanying guided-inquiry questions: carbohydrate structures, bacterial digestion, and fermentation products. In the carbohydrate structures section, students built models of amylose, amylopectin, or cellulose (Figures 1C–E) using either the K’nex pieces (in-person) or digital playground document (remote), following the kit instructions document described above, to explore the specific types of glycosidic bonds present in the biochemical structure of each carbohydrate. In the bacterial digestion section, students used the card deck to explore the various microbes in the gut and the specific glycosidic bonds they break to digest different carbohydrates. The bacterial digestion section also included two “apply your understanding” questions, which represented the final stage of the learning cycle (Abraham, 2005; Eberlein et al., 2008; Cracolice, 2009) where students had to apply their newly formed knowledge to a new context. Finally, in the fermentation products portion of part 1, students read a brief introduction to SCFAs and responded to a multi-part “apply your understanding” question. Part 2 of the guided inquiry activity did not require the kit and consisted of two parts: effects on the host and case study. In the effects on the host section, students interpreted a figure and answered questions about the benefits and drawbacks of three SCFAs – acetate, propionate, and butyrate. In the case study section, students interpreted two figures from primary literature sources (David et al., 2014; Greene et al., 2020) and responded to application questions.

All documents referred to above are available at: https://stembuild.ncsu.edu/lesson-plan/bacterial-fermentation/. The key for the guided inquiry worksheet can be made available upon request.

### 2.3. Implementation of the tactile teaching tool-guided inquiry activity

**Figure 2:**
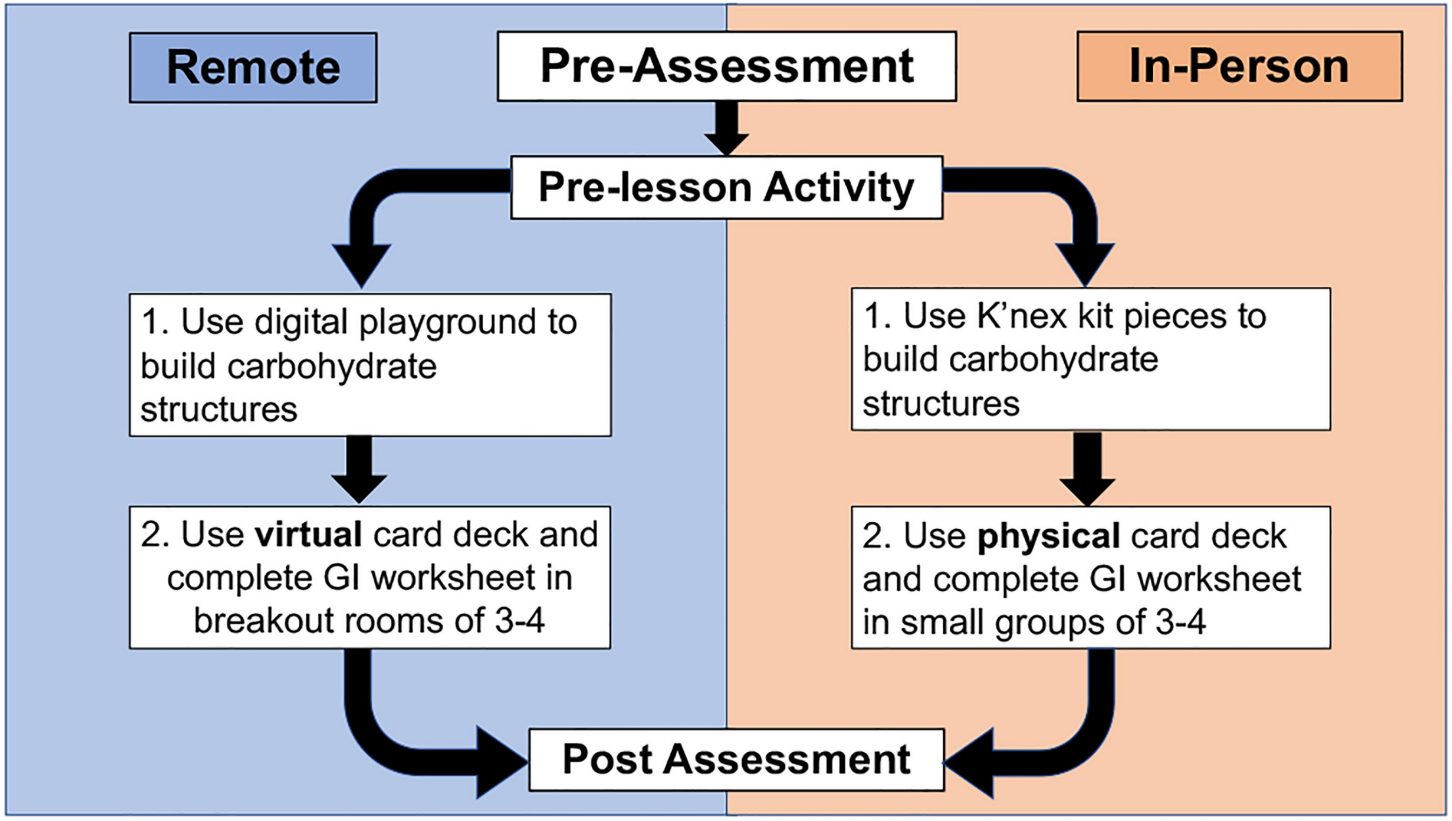
Schematic of how this TTT-GI activity was implemented in both in-person and remote educational settings. All students completed the same pre-assessment, pre-lesson activity, and post-assessment.

Figure 2 depicts a schematic of how this TTT-GI activity was implemented for both in-person and remote learners. For the pre-lesson activity, all students (remote and in-person learners) completed a preassigned reading (Stevens and Hume, 1998) and a GRQ that they were required to submit before class *via* the course LMS. The goal of this pre-lesson work was to familiarize students with the concepts they would explore further during the in-class portion of the activity. Students also watched a pre-lesson video that walked them through a primary literature source (Leshem et al., 2020), which prompted them to evaluate and reflect on how diet impacts the gut microbiome. These pre-lesson components of the activity can be found at: https://stembuild.ncsu.edu/lesson-plan/bacterial-fermentation/.

At the start of the class period when the TTT-GI activity was implemented, the instructor led a brief class discussion encouraging students to think about how microbes might digest different diets. All students, remote and in-person, participated in this discussion. Afterwards, students worked in groups to complete the guided-inquiry activity. Students who attended in-person formed groups of 3–4, with one physical kit per group. Students who attended remotely were put into Zoom breakout rooms consisting of 3–4 students and instructed to download the remote digital playground and card deck documents that were posted to the course LMS. All students had access to the kit instructions and guided inquiry documents, which were also posted to the course LMS. Student teams first worked through part 1 of the activity while manipulating their kit either in-person or virtually. After completing part 1, the entire class regrouped to review the three “apply your understanding” questions in part 1 of the guided-inquiry document. Slides used for the class session are available at: https://stembuild.ncsu.edu/lesson-plan/bacterial-fermentation/.

Afterwards, groups re-formed to complete part 2 of the activity. Student groups in the 75-min section of the class completed part 2 of activity in the same class period as part 1 and then re-grouped as an entire class to review responses. Student groups in the 50-min section started part 2 but did not complete it in class and were told to complete the questions on their own time. The instructor spent approximately 10 min at the start of the next class reviewing the case study questions with the 50-min section. A key for the guided-inquiry document was posted to the LMS for both sections after the activity was completed for students to review (available upon request). Students in the 50-min section also had a non-mandatory session to review the activity content, which was facilitated by one of the undergraduate peer instructors. This session was recorded and posted to the course LMS. Students in the 75-min section did not have a dedicated peer instructional review session on this activity or access to the recording, although they could discuss aspects of this lesson with the peer instructors during their virtual sessions.

### 2.4. Data collection

Students completed a pre-assessment *via* Qualtrics during the first week of the semester. This pre-assessment included the 9 assessment questions (Table 1) as well as a voluntary demographic questionnaire (Supplementary material 1). Representative assessment questions can be found in Supplementary material 6. The demographics survey asked students to self-report their race and ethnicity, gender identity, transfer status, educational level, first-generation college student status, disability status, prior coursework completed, and degree plan. To limit the chances of stereotype threat (Steele and Aronson, 1995; Steele, 1997; Spencer et al., 1999), the demographics questionnaire was presented after the pre-assessment questions and participants could choose to not answer a question and/or denote “prefer not to respond.”

At the start of the first class session where the TTT-GI activity was implemented, students self-reported their mode of attendance using in-class polling software (Learning Catalytics). Students who were in-person were defined as students being present in the physical classroom at the time this question was deployed. Students who were remote were defined as students who were attending class virtually (synchronous) *via* a live stream Zoom link at the time this question was deployed.

The post-assessment comprised the same questions as the pre-assessment. Assessment questions 1 and 7 were assigned for online homework approximately 2 weeks after the activity was implemented. Students had 45 min to complete this homework assignment, which consisted of 15 questions related to four lessons within the class, *via* the course LMS. The remaining assessment questions were on the final exam, approximately 3 weeks after implementation of the activity. The final exam was a proctored paper exam consisting of 50 multiple choice questions assessing content from 7 individual lessons across the final unit and students had 3 h to complete it. Refer to Supplementary material 6 for representative assessment questions.

### 2.5. Data analysis

A total of 182 students both consented to the study and completed both the pre-and post-assessments. Student identifiers were removed from both the assessment and demographics data and replaced with a unique study identifier. To analyze the demographics data, we binned the variable responses and determined the number of individuals, as well as percentage of the total population, for each bin. We used Asai’s persons excluded due to ethnicity or race (PEER) framework to bin the race and ethnicity demographic variable (Asai, 2020). We defined educational level based on the number of credit hours the student had completed. Life science degree plans referred to students who reported the following majors: biochemistry (B.S.), biology (B.S. or B.A.), psychology and neuroscience (B.S.), and exercise and sports science (B.A.). Pre-health degree plans referred to students who reported the following majors: pre-nursing, pre-nutrition, pre-health, pre-pharmacy, pre-dental, pre-physician’s assistant.

To analyze performance on individual assessment questions on both the pre-and post-assessment, correct responses received a “1” and incorrect responses received a “0.” We calculated the average and standard deviation of the pre-and post-assessment scores for each question. A paired *t*-test (*p* < 0.05) was performed for each assessment question using GraphPad Prism (version 9) to compare the pre-and post-assessment scores for each question.

To determine learning gains of the activity, we subtracted the sum of students’ pre-assessment scores for all nine questions from the sum of their post-assessment scores. We used two statistical analyses to evaluate differences between student populations. First, we performed an unpaired Student’s *t*-test (*p* < 0.05) to determine if there were statistical differences between the gains in different student populations. These analyses were performed in GraphPad Prism (version 9). We then determined the effect size (Hedges’ g) of the TTT-GI activity on specific student populations using Microsoft Excel. For our analysis, Hedges’ g values of 0.3 or below were small effect sizes; Hedges’ g values between 0.31 and 0.70 were medium effect sizes; Hedges’ g values greater than 0.71 were large effect sizes.

## 3. Results

### 3.1. Description of the student population

The descriptive statistics of the student population participating in this study are outlined in Table 2 and Supplementary Table 1. Most students (*n* = 158) were direct entry students, with 91 of those students completing high school with some college credit. The remaining students were transfer students from either a 2-year (*n* = 10) or 4-year (*n* = 14) institution. The student population consisted primarily of 2^nd^ -and 3^rd^-year students (*n* = 148) as compared to 1^st^- and 4^th^-year students (*n* = 30). Four students were either non-degree seeking students or enrolled in some form of non-traditional degree program. Eleven of the 182 students declared one or more self-reported disabilities. Using Asai’s (2020) PEER framework, 42 students identified as PEER and 139 students identified as non-PEER. Most students (*n* = 140) had at least one parent who had completed a bachelor’s degree (non-first generation college student) and the population consisted of 92 life science majors and 89 pre-health majors. Finally, 126 students had previous experience with a 200-level biology course at the institution, whereas 56 had only completed the pre-requisite, 100-level, introductory biology course at either the same institution or elsewhere.

**Table 2:** Self-reported demographic variables for the students who participated in the TTT-GI activity.

Category	Subcategory	N	%
Race/ethnicity			
	PEER	42	23.2%
	Non-PEER	139	76.8%
	Did not disclose	1	0.5%
Gender identity			
	Female	120	65.9%
	Male	56	30.8%
	Non-binary	6	3.3%
Transfer status			
	Transferred from 2-year institution	10	5.5%
	Transferred from 4-year institution	14	7.7%
	Direct entry without college credit	67	36.8%
	Direct entry with college credit in HS	91	50.0%
Educational level			
	First year student (0–30 credits)	15	8.2%
	2^nd^ year student (31–60 credits)	95	52.3%
	3^rd^ year student (61–90 credits)	53	29.1%
	4^th^ year student (91–120 credits)	15	8.2%
	Other	4	2.2%
First generation status			
	First generation college student	40	22.0%
	Not a first-generation college student	140	76.9%
	Unknown	2	1.1%
Disability status			
	Disabled	11	6.3%
	Abled bodied	163	93.7%
	Did not disclose	8	4.4%
Previous coursework			
	Intro biology	56	30.8%
	Upper-level biology coursework	126	69.2%
Class meeting time			
	50 min	90	49.5%
	75 min	92	50.5%
Degree plan			
	Life sciences	92	49.2%
	Pre-health	89	50.8%
	Other	1	0.5%

The total population was 182 individuals.

### 3.2. Global assessment of student learning outcomes

**Figure 3:**
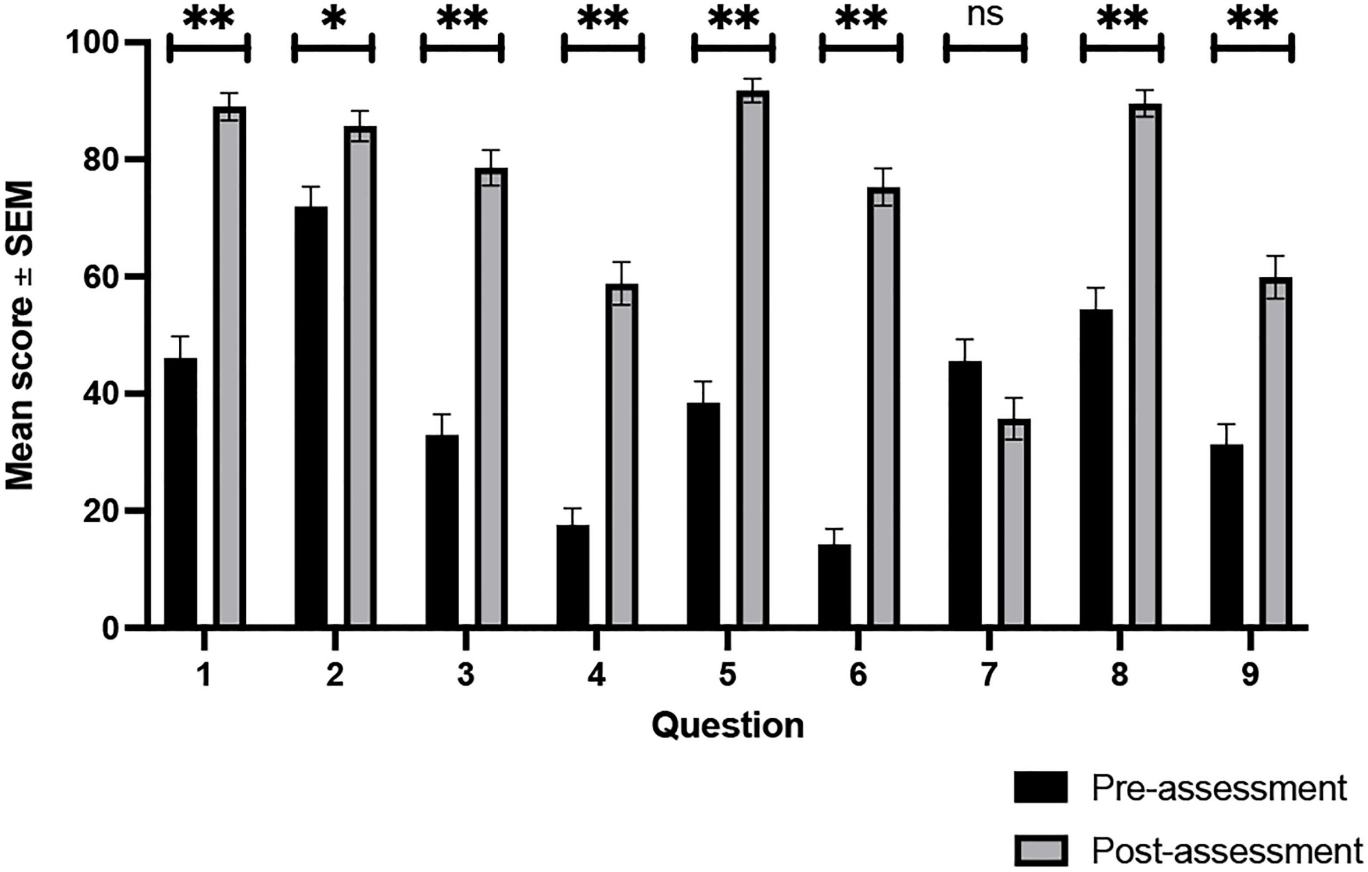
Comparison of pre-assessment and post-assessment scores for each question. The pre- assessment and post-assessment percentage scores (mean ± SEM) for each question from a total of 182 students are depicted. Data were analyzed using a paired *t*-test. * Denotes *p* < 0.05; ** denotes *p* < 0.001; ns denotes not significant.

We addressed our first research question by comparing the scores on pre-and post-questions that assessed the three learning objectives of this TTT-GI activity (Table 1). As shown in Figure 3 and Supplementary Table 2, we observed statistically significant gains in eight of the nine questions when comparing the pre-and post-assessment scores. These eight questions were associated with LO1 (questions 1, 5, and 8), LO2 (question 4), and LO3 (questions 2, 3, 5, 6, 7, and 9) and demonstrate that students attained the learning objectives for this activity. Of note, we observed gains of 40% or more for questions 1, 3, 4, 5, and 6, on the post-assessment compared to the pre-assessment. For question 7, which assessed LO3, we observed a mean post-assessment score that was less than the pre-assessment score. Given that students made significant gains for all other questions assessing LO3, we suspect that the decrease in scores for question 7 has to do with a flaw in the design or implementation of the question.

### 3.3. Activity outcomes based on mode of student attendance

To address our second research question, we evaluated if there were differences in gains on the assessment based on students’ mode of class attendance. As shown in Table 3, most students attended in-person (*n* = 96) and saw average learning gains of 36.10 ± 22.69%. Students who attended remotely (*n* = 60) had average learning gains of 29.44 ± 28.26%. Hedges’ g calculations revealed that in-person students had a small effect size, or minimal and insignificant increase in learning gain compared to remote students (Table 3). It should be noted that there were 26 students, whose mode of attendance was unknown, who had average learning gains of 40.61 ± 22.89%. This learning gain difference was nonsignificant compared to either the in-person (*p* = 0.372) or remote (*p* = 0.079) student populations.

**Table 3:** Effect size for mode of student attendance and class section.

Population		N	Gains (%)	Hedges’ g	*p*-Value
Class format					
	In-person attendance	96	36.10 ± 22.69	0.267	0.107
	Remote attendance	60	29.44 ± 28.26		
Class section enrolled					
	75-min class	92	29.95 ± 26.14	−0.380	*0.011
	50-min class	90	39.26 ± 22.67		

Learning gains represent the average pre-assessment score (%) subtracted from the post-assessment score (%) for all nine assessment questions. Student learning gains for each category was analyzed using Hedges’ g and Student’s *t*-test (*p* < 0.05), with * denoting statistical significance. The second of the two variables in the table served as the control in the Hedges’ g calculations.

Interestingly, we found an intermediate effect size (Hedges’ g = −0.380) for students who attended the 75-min section compared to the 50-min section (Table 3). Students in the 75-min section had average learning gains of 29.05 ± 26.14%, whereas students in the 50-min section had average learning gains of 39.26 ± 22.67%. This difference in learning gains between the two sections was significant, demonstrating that the students in the 50-min section had greater learning gains than students in the 75-min section. It should be noted that the population of the two sections was the same size, with the 75-min section having 92 individuals and the 50-min section having 90 individuals (Table 3).

### 3.4. Impact of activity on specific student populations

To address our final research question, we calculated Hedges’ g to evaluate the effect sizes of the learning gains for this TTT-GI activity between different student populations (Table 4; Supplementary Table 3). First, compared to direct entry students, transfer students from 2-year or 4-year institutions had Hedges’ g of −0.337, an intermediate effect size, although there was a nonsignificant difference between these populations. Compared to upper-division students, first year students had intermediate to high effect sizes (−0.659 to −1.023) that were associated with significantly lower learning gains (Table 4; Supplementary Table 3). This demonstrates that while there is a modest and nonsignificant difference in learning gains between transfer and direct entry students, first-year students had significantly lower learning gains associated with this activity than second-, third-, or fourth-year students.

**Table 4:** Effect size of activity on diverse student populations.

Population	Comparison	Hedges’ g	*p*-Value
Transfer status			
	Transfer (2−/4-year) vs. direct entry	−0.337	0.126
Educational level			
	First year vs. second year students	−0.659	*0.020
	First year vs. third year students	−0.702	*0.019
	First year vs. fourth year students	−1.023	*0.009
Racial/ethnic identity			
	PEER vs. Non-PEER	−0.260	0.142
Gender identity			
	Women vs. Men	−0.173	0.2779
First generation status			
	First generation vs. non-first-generation students	0.133	0.474
Disability status			
	One or more disability vs. no disability	0.273	0.382
Pre-requisite coursework			
	Upper-level Biology vs. Intro Biology coursework completed	0.448	*0.006
Degree plans			
	Life science vs. pre-health majors	0.055	0.700

Student learning gains for different student populations were analyzed using Hedges’ g and Student’s *t*-test (*p* < 0.05), with * denoting statistical significance. The second demographic group in each comparison serves as the control.

We observed a small effect size (Hedges’ g = −0.260) for students who identified as PEER compared to non-PEERs (Table 4; Asai, 2020), with non-PEER students having slightly higher learning gains than PEER students (Supplementary Table 3). These differences, however, were nonsignificant (Table 4). The learning gains effect size for individuals who identified as women was small (Hedges’ g = −0.173) compared to those who identified as men, with men having nonsignificant but slightly higher learning gains than women (Table 4; Supplementary Table 3). We also observed nonsignificant differences in learning gains for individuals who identified as non-binary (*n* = 3) compared to both women (*p* = 0.419) and men (*p* = 0.642; Supplementary Table 3). These results demonstrate that while there are slightly lower learning gains for students underrepresented in STEM [National Center for Science and Engineering Statistics (NCSES) and Directorate for Social, Behavioral and Economic Sciences, 2021], specifically PEERs and women, the differences in learning gains for these populations are small and nonsignificant.

Interestingly, however, we observed a small effect size (Hedges’ g = 0.133) for students who were first-generation compared to those who had at least one parent complete a bachelor’s degree (non-first generation). The difference in learning gains between these populations were also nonsignificant, with first-generation students having slightly higher learning gains (Supplementary Table 3). We also observed a small, yet positive, effect size for students who self-reported one or more disabilities compared to students with no reported disability (Hedges’ g = 0.273; Table 4). As demonstrated in Supplementary Table 3, students with at least one reported disability had slightly higher, yet nonsignificant, learning gains compared to their abled bodied peers. These data suggest that this TTT-GI approach may be particularly beneficial for students with disabilities and/or first-generation students, populations of students traditionally underrepresented in STEM [National Center for Science and Engineering Statistics (NCSES) and Directorate for Social, Behavioral and Economic Sciences, 2021].

As shown in Table 4, intermediate effect sizes were found for students who had completed 200-level or higher biology courses (Hedges’ g = 0.448) compared to those who had only completed the 100-level intro biology pre-requisite course. Specifically, students who had completed upper-level biology coursework (200-level or higher) had significantly greater learning gains than those who had only completed the 100-level prerequisite course necessary for enrollment in this Human Anatomy and Physiology course (*p* = 0.006). While clearly this previous course enrollment impacted student learning gains associated with this TTT-GI activity (Supplementary Table 3), degree plans did not appear to impact student learning gains given that we calculated a small effect size (Hedges’ g = 0.055) for students who were life science majors compared to pre-health majors (Table 4; Supplementary Table 3). Thus, if students had completed at least one previous 200-level biology course, their learning from this TTT-GI activity appeared to be positively impacted regardless of their degree plan.

Because we found an intermediate effect size for students who had completed a 200-level or higher biology course having higher learning gains from this TTT-GI activity, we were interested in exploring the completion rates of these upper-level courses amongst diverse students in our sample. As shown in Supplementary Table 4, we found discrepancies in diverse student populations’ completion of upper-level biology coursework. Transfer students, first year students, PEER students, women, first-generation students, and pre-health students had lower completion rates of 200-level biology coursework or higher compared to their peers. For some demographic variables, such as race or ethnic identity, there was a 40% difference in the completion of upper-level biology courses between PEER and non-PEER students. As expected, there was also a strong difference (greater than 65%) in completion rates for upper-level biology courses when comparing first year students and students from all other educational levels. Interestingly, there was no difference in upper-level biology coursework completion for students who attended remote versus those that attended in-person, although a greater percentage of students with unknown attendance did complete upper-level coursework (Supplementary Table 4). We also found that there was a greater percentage of students in the 50-min class who completed upper-level biology coursework compared to those in the 75-min section (Supplementary Table 1).

## 4. Discussion

Tactile teaching tools with guided inquiry (TTT-GI) approaches have been previously described to promote student learning of complex biological topics (Ramirez and Gordy, 2020). Designed with principles of Universal Design for Learning in mind, these TTT-GI approaches have the potential to provide inclusive and equitable learning experiences for diverse learners (Rose and Meyer, 2006; Burgstahler and Corey, 2008; Rappolt-Schlichtmann et al., 2012; Meyer et al., 2014; Hasper et al., 2015). We developed a novel TTT-GI activity to help students in a 200-level, large-enrollment, Human Anatomy and Physiology course to learn how gut microbes affect host health by digesting and fermenting carbohydrates to produce SCFAs. This activity was designed for a hyflex learning environment, where students could build amylose, amylopectin, and cellulose either by using physical K’nex pieces in-person or by manipulating digital versions remotely. All students completed the same guided-learning activity and the learning objectives (Table 1) of the activity were assessed using 9 questions that were implemented in a pre−/post-assessment scheme. Given the novelty of this active learning activity, we were therefore interested in evaluating the impact of this TTT-GI module on student learning. Herein, we discuss our interpretation of the three research questions in the context of our collected data and previous research, while also providing limitations of our approach and possible modifications to this TTT-GI activity.

### 4.1. Impact of the activity on student learning outcomes

We first sought to determine if students attained the learning objectives (Table 1) for this TTT-GI activity. For every question but one, students made significant gains on the post-assessment compared to the pre-assessment (Figure 3; Supplementary Table 2). Given that these eight questions each assessed one or more of the three learning objectives of this activity, we conclude that students did attain the learning objectives as intended. Notably, two of these learning objectives (LO1 and LO3) align to higher-order cognitive domains, specifically analyze and evaluate (Table 1). This demonstrates that our TTT-GI approach can be used to develop students’ higher-order cognitive skills, which are necessary for modern careers within biology (Brewer and Smith, 2010). Our results also demonstrate that this activity provides a novel and effective approach to teach concepts of the gut microbiome. Most of the previous descriptions of gut microbiome activities were in laboratory, microbiology or other molecular biology courses (e.g., Estes, 2015; Wang et al., 2015; Robertson-Albertyn et al., 2016; Coil et al., 2017; Lentz et al., 2017; Goller and Ott, 2020). By contrast, here we present an effective way to include the physiological impacts of the gut microbiome using a TTT-GI approach within an undergraduate Human Anatomy and Physiology course. This is important for instructors to consider when designing human or comparative Anatomy and Physiology courses, given recent advancements in our understanding of how the gut microbiome impacts the physiology of the host (Turnbaugh et al., 2007; Baquero and Nombela, 2012; Pilla and Suchodolski, 2020).

On our assessment we found that question 7, which assessed LO3, had lower post-assessment scores than the pre-assessment (Figure 3). We suspect that this could be due to several factors. First, while this question assessed a higher order learning objective, the question itself required memorization of the specific fermentation products of a bacterial taxon to be able to predict the physiological consequence of the SCFA. While we typically associate rote-memorization with lower-performing students (Grove and Bretz, 2012; McGuire, 2015; Liao et al., 2019), previous research has also shown that students adjust their learning to reflect the perceived cognitive level of the assessment (Thiede et al., 2011; Jensen et al., 2014). The Human Anatomy and Physiology course that implemented this TTT-GI had stressed learning objectives and higher-order Bloom’s taxonomic levels (Anderson and Krathwohl, 2001) throughout the duration of the course and most questions on previous formative and summative assessments required higher-order cognitive skills. It is therefore possible that because question 7 aligned to a higher-order learning objective, students performed poorly on this question because they were expecting a question that required higher-order strategy and not the rote-memorization that is typically associated with lower-level cognitive domains. Another factor that could have resulted in decreased post-assessment scores for question 7 is that this question was on the students’ homework assignment and not on the final exam, where most of the other post-assessment questions were found. Our results may therefore reflect either that students had less time to study the material to demonstrate their learning associated with this question, or that the low-stakes homework assignment was not prioritized to the same extent as the high-stakes final exam. It should be noted, however, that the homework assignment that had question 7 also included question 1, for which we observed significant learning gains (Figure 3). Additionally, this homework assignment was open-note and the digital card deck was available for students to access on the LMS during the homework assignment, which should have given students insight into how to correctly answer this question.

### 4.2. Equivalent student gains for those attending in-person versus remotely

As shown in Table 3, there were minimal differences in student learning gains for students who engaged with this TTT-GI activity in-person versus remotely. We suspect that this is due to the intentional adaptation of the in-person activity for remote learners. While in-person learners interacted with a physical kit consisting of K’nex pieces and card deck, remote students had the opportunity to interact with kit components digitally *via* the digital playground and card deck (available at: https://stembuild.ncsu.edu/lesson-plan/bacterial-fermentation/). Both in-person and remote learners completed the same guided-inquiry activity. This provides further evidence that active learning can be adapted for and impactful in remote environments, which was required of instructors throughout the COVID-19 pandemic (Morrison et al., 2021; Nguyen et al., 2021; Rossi et al., 2021; Venton and Pompano, 2021). We did not track the daily mode of attendance (i.e., in-person vs. remote) per individual across the semester. A future longitudinal study could assess the impacts of attending class in-person vs. remotely on students’ academic performance and learning gains.

Our populations of remote and in-person participants did not differ in the number of students who had completed upper-level (i.e., 200-level or above) biology course-work versus those who did not (Supplementary Table 4). That said, students who attended remotely achieved slightly lower learning gains compared to their in-person peers, which could be due to a variety of confounding factors. The remote option was encouraged for students who faced challenges related to the ongoing COVID-19 pandemic, suggesting that factors beyond this specific TTT-GI activity may have contributed to their nonsignificant lower learning gains. These challenges include being diagnosed with and/or exposed to SARS-CoV-2, having to care for others, attending class remotely in a distracting environment, and the increased socio-economic burden placed on many students because of the pandemic (Adedoyin and Soykan, 2020; Onwuegbuzie et al., 2020; Kee, 2021; Wester et al., 2021). It is also important to highlight the numerous mental health challenges that many students faced during the pandemic, which may also have impacted their academic outcomes during remote instruction (Onwuegbuzie et al., 2020; Kee, 2021; Lemay et al., 2021; Wasil et al., 2021). It is unclear to what extent the students in our study were impacted by these specific pandemic-associated factors, or how these challenges may have contributed to remote students’ lower learning gains. This is an area that warrants further investigation.

Another factor that may have contributed to the lower learning gains for remote students is that this TTT-GI activity was designed to include cooperative learning pedagogies (Johnson et al., 1991, 2010), for which students work in collaborative teams to explore a particular topic. Interestingly, challenges associated with group work and/or academic engagement in a remote environment, particularly throughout the pandemic, have been previously described (Wester et al., 2021; Wildman et al., 2021). In some cases, this lack of engagement can result in students developing negative views towards their STEM courses (Wester et al., 2021). Anecdotally, while we were able to observe group interactions for students who participated in class, we observed minimal group interactions in the Zoom breakout rooms for remote students. It is unclear why students in the remote setting were not interacting with each other similar to in-person students, but we suspect that remote students may have chosen to work on the activity individually in the breakout rooms instead of collaboratively, as intended. This may have negatively contributed to their academic engagement with the activity and thus resulted in lower learning gains (Table 3). It is therefore important for us to further investigate the importance of the cooperative learning (Johnson and Johnson, 1989; Johnson et al., 1991, 2010) aspect of the activity and how structured teams promote student learning through a TTT-GI approach.

We observed the highest learning gains across the three attendance groups in the students with unknown attendance. We suspect that this group could simply be students who came to class late, either in-person or remotely, and missed the poll question that queried their mode of attendance. Upon further review of this student population, 80.77% of students with unknown attendance had completed upper-level biology courses beyond intro biology (Supplementary Table 4).We know that highly motivated and experienced students tend to be high-achieving (Saenz et al., 1999; Trevino and DeFreitas, 2014; Ribeiro et al., 2019; Steinmayr et al., 2019) and it is therefore possible that students in this unknown mode of attendance group would have done well regardless of their ability to interact with the tactile teaching tool and/or guided inquiry activity. Additionally, it has been shown that group activities, especially in introductory biology courses, strongly impact high achieving students (Marbach-Ad et al., 2016). This could explain why these students with unknown attendance had higher learning gains, as our data (Table 4) revealed that experience with upper-level biology coursework correlated with increased learning gains for our activity.

### 4.3. Differential learning gains for TTT-GI activity based on length of class section

Although the 50-min section had less time in class to interact with the activity, we observed significantly increased learning gains in this population compared to the students enrolled in the 75-min section (Table 3). Survey responses revealed that a slightly larger percentage of students in the 50-min section had completed upper-level biology coursework compared to students in the 75-min section (Supplementary Table 1), which could explain our findings. That said, other factors may explain why students in the 50-min section had higher learning gains than the other section. First, students in the 50-min section were given the option to attend a review session that focused on the learning objectives of the activity that was facilitated by a peer instructor. While only 10 students were reported to have attended this session, the session was recorded and posted to the course LMS for others to review. It is unclear how many students within the 50-min section reviewed this recording and how this resource may have impacted the learning gains for the 50-min section compared to the 75-min section, which did not have a dedicated review session for this lesson. That said, students in the 75-min section did have the opportunity to discuss the lesson informally with a peer instructor. Data specifying which students accessed the recording and/or specifically discussed this lesson with a peer instructor are unavailable.

Another factor that may explain why the 50-min section had higher learning gains is the inherent difference in cognitive load between the two sections. For the 75-min section, the entirety of the activity was presented in a single class period with no additional review. Students in the 75-min class, while having more contact time with the activity, could have experienced a higher cognitive load, which may have diminished their gains. In this highly structured activity, students were presented with a scaffold of what would be expected during the activity and were allowed to work through each section before the class re-grouped for instructor-led review. It has been shown that this method is highly effective for teaching inquiry learning-based activities by providing a scaffold to support student learning goals and reduce their cognitive load (Hmelo-Silver et al., 2007). Compared to the 75-min, the cognitive load of the 50-min section was likely further reduced by the additional review of part 2 of the guided-inquiry activity, for which students critically evaluated two figures from a primary literature source at the start of the subsequent class session. This spaced learning effect has been shown to reduce the cognitive load on individuals and promote more significant learning, as the working memory is given time to process and store the information (Chen et al., 2018). A spaced learning approach increases neurogenesis within the regions of the brain that are responsible for learning and memory acquisition (Sisti et al., 2007), and could explain the significant increase in learning gains observed in students enrolled in the 50-min section compared to the 75-min section (Table 3). Together, this information suggests that it may be beneficial to break our TTT-GI activity into two separate class periods to reduce the cognitive load of students, instead of presenting it in a single class session.

### 4.4. TTT-GI activity is particularly beneficial for upper-division students with advanced biology coursework experience

First-year students typically struggle with the transition from secondary to collegiate level studies, especially those that identify as PEER (Briggs et al., 2012). As expected, among the four academic years, first-year students experienced the lowest learning gains (Table 4). We suspect this is due to their relative inexperience with the collegiate environment, study habits, and general lack of experience with upper-level coursework. The TTT-GI approach may represent an activity most first-year students likely have not experienced previously due to the lack of these types of active learning opportunities in high school and/or multiple semesters of online learning during the COVID-19 pandemic. This is not to say that online learning is inherently less effective, but rather to acknowledge the learning losses associated with broader cognitive deficits experienced by students across the pandemic, as well as the loss of opportunities to build communities or a sense of belonging, which can be lost (or at least diminished) when students attend class remotely. Thus, our observation that first-year students had lower learning gains than other students may result from unfamiliarity with these types of active learning modules in the college setting.

One challenge with interpreting these results is that students may have self-reported an educational level higher or lower than their actual time at the university, given that we defined educational level by the number of credit hours completed. A student in their first year at the institution, for example, could potentially matriculate with enough credits to classify themselves as second-year student, which could skew the number of students in each group (Table 2). In the future we could revise the survey to have students specify the number of credits they have completed while at the current R1 institution, versus transfer credits (i.e., from other 2- or 4-year institutions, high school etc.). That said, in the current study upper-division students overall had greater learning gains than lower-division students. This observation could be attributed to upper-division students having more experience with learning at the university level. Our observations suggest that if this activity were to be implemented in a primarily first-year course, revisions would likely be needed to accommodate these students.

Another explanation for our observation that first-year students had lower learning gains than upper-level students is that most first-year students had only previously completed the 100-level introductory biology pre-requisite for this course and did not have experience with advanced (200-level or above) biology coursework (Supplementary Table 3). Previous coursework in chemistry and biology increases the rate of students passing Human Anatomy and Physiology courses (Hull et al., 2016), and previous experience within higher education predicts student academic success in STEM disciplines (Rogaten and Rienties, 2018). As shown in Table 4, previous enrollment in a 200-level biology course had a medium effect size, which correlated to significantly higher learning gains compared to students who had previously only completed the 100-level pre-requisite.

The obvious explanation for our observations is that the concepts and skills needed for this particular TTT-GI activity are developed in an advanced (200-level or higher) biology course. For example, the topic of the physiological impacts of the gut microbiome was not covered in the textbook used for the course and required students to read scholarly literature sources (Stevens and Hume, 1998; Leshem et al., 2020) as their pre-reading assignment instead. Being able to read scholarly literature is a skill that is developed and fostered throughout students’ undergraduate experience (Levine, 2001; Kuldell, 2003; Brewer and Smith, 2010), and it is likely that students who completed at least one 200-level biology course had previous experience reading scholarly literature, whereas students who completed only the 100-level intro biology prerequisite did not. Alternately, topics related to this TTT-GI activity, such as the gut microbiome, carbohydrate structures, and glycolysis and fermentation pathways, may have been covered in other 200-level courses, thus providing previous exposure for this population of students. It should be noted that the prerequisite 100-level biology course that is taught at the same institution does have a lesson dedicated to the microbiome and other lessons in the class focused on necessary prerequisite knowledge (e.g., carbohydrate structures and cellular respiration pathways). That said, not all students at the institution take this 100-level biology course, as they often have either AP credit from high school or credit from another institution that fulfills this prerequisite. Students who transfer from one institution to another may have lower learning gains associated with specific learning objectives (Whitfield, 2005; Hoffman et al., 2016; Corwin et al., 2019), likely resulting from differences in how comparable courses are taught at differing institutions. Therefore, some of the students who had only completed the 100-level prerequisite biology course could have been at a further disadvantage if they had not been exposed to some of the concepts in this TTT-GI activity previously. Finally, the impact of the COVID-19 pandemic on learning may also explain our observation that students with 200-level biology had greater learning gains. Studies have shown reduced student engagement, including class participation and interactions with peers and faculty, during the COVID-19 pandemic (Wester et al., 2021), while student engagement activities positively correlate to academic outcomes (Fredricks et al., 2004; Handelsman et al., 2005; Rocca, 2010; Gasiewski et al., 2012; Olson and Riordan, 2012). It is therefore possible that students in our sample who completed biology coursework during a remote COVID semester may have experienced differences in learning, which may have contributed to their decreased gains for this TTT-GI activity. Since this study was conducted in the spring of 2022, after a semester of in-person learning, students who had taken the 100-level introductory biology prerequisite in the previous fully remote academic year may have had a greater disadvantage due to both the impacts of the pandemic on their learning and the time distance between enrollment in the prerequisite introductory biology course and this Human Anatomy and Physiology course. By contrast, students who had completed at least one 200-level course had the chance to reinforce their learning from prior coursework, which may have benefitted them on this TTT-GI activity.

### 4.5. Impact of TTT-GI activity on diverse student populations

Active learning in STEM classrooms positively impacts diverse student populations. For instance, Beichner et al. (2007) used a SCALE-UP approach instead of traditional lecture to teach a calculus-based engineering course, resulting in higher success rates for all students, but particularly for females and minorities. Similarly, a TTT-GI activity used to teach introductory biology students about the *lac* operon resulted in greater learning gains for students at a rural minority-serving institution as compared to a non-minority serving public R1 institution (Gordy et al., 2020). Finally, Theobald et al. (2020) found that active learning approaches benefited all students, but disproportionately benefited students from underrepresented groups, particularly minority students and students from lower socioeconomic backgrounds. We therefore wanted to determine how the learning of diverse student populations was impacted by this gut microbiome TTT-GI activity.

While transfer students had lower learning gains than direct entry students (Supplementary Table 3), this was a nonsignificant difference (Table 4). This finding was surprising considering that lack of engagement within the classroom, such as opportunities for active and collaborative learning, is a major hindrance to academic success for transfer students (Kuh, 2003). While our observations may be explained by the low representation of transfer students within our population (*n* = 24) and/or many of the transfer students not completing at least one upper-level biology course (Supplementary Table 4), our results may also reflect unique aspects of the Human Anatomy and Physiology class where this TTT-GI activity was implemented. Specifically, this course was a high-enrollment course that may be unfamiliar to many transfer students, particularly from 2-year institutions. Previous research has shown that transfer students have difficulties transitioning into high-enrollment courses compared to the small course sizes at 2-year institutions (Townsend and Wilson, 2006). Additionally, while transfer students may have completed the prerequisite coursework for upper-level courses in their major, they may not have achieved the necessary prerequisite knowledge based on differences in how the prerequisite courses at pre-transfer institutions are taught (Hoffman et al., 2016; Corwin et al., 2019). However, since we observed nonsignificant differences between transfer and direct entry students, we suspect that our TTT-GI approach has the potential to benefit both groups of learners, with additional research needed to evaluate how this approach specifically impacts students who transfer.

Considering the mounting evidence that active learning pedagogies are especially efficacious for historically underrepresented populations in STEM (Beichner et al., 2007; Gordy et al., 2020; Kressler and Kressler, 2020; Theobald et al., 2020), the finding that PEER and women students experienced lower learning gains from this TTT-GI activity was also somewhat surprising (Table 4; Supplementary Table 3). While the effect size is small for PEER vs. Non-PEER students, our results may be explained by our student population comprising only 23.2% PEER students and thus representing a small sub-population within our larger sample (Table 2). That said, both PEER students and women had lower rates of previous experience with upper-level biology courses than non-PEER students or men, respectively (Supplementary Table 4). We suspect that this may be particularly the case for women, who comprised 65.9% (Table 2) of our student population. As discussed above, prior enrollment in 200-level biology courses resulted in significantly greater learning gains than students who had only completed the prerequisite 100-level biology course. It is therefore possible that the slightly lower learning gains made by PEER students and women reflect previous biology course experience. Regardless, further research is needed to explore how our TTT-GI activity impacts learning in both PEER and female students. Our findings also highlight the importance of proactive and holistic academic advising for diverse student populations to help them achieve success in their coursework (Smith and Allen, 2006; Museus, 2021).

Two populations that experienced learning gains were first-generation students and students who reported one or more disabilities (Table 4; Supplementary Table 3). The TTT-GI approach was designed using principles of Universal Design to provide equitable learning experiences for all students, including those with disabilities (Gordy et al., 2020; Ramirez and Gordy, 2020). While not significant, we did observe a trend of higher learning gains for first-generation students and students with one or more disabilities (Table 4; Supplementary Table 3). This was an exciting finding for us, as it provides preliminary evidence that our TTT-GI approach may benefit students historically underrepresented in STEM, as observed with other active learning approaches (Beichner et al., 2007; Gordy et al., 2020; Theobald et al., 2020). Further, while we found no difference in upper-level biology coursework completion between students with one or more reported disabilities and those with no disabilities, we did detect greater than 19% reduction in upper-level biology completion rates amongst first generation students compared to their peers (Supplementary Table 4). This may suggest that our TTT-GI activity is particularly beneficial for first generation students, which mirrors findings from other studies (Filkins and Doyle, 2002). We are cautious, however, to make major conclusions from these findings given the small sample size of students who self-reported being first-generation (*n* = 40) or having one or more disabilities (*n* = 11) in our sample (Table 2). Further research is needed to investigate how this TTT-GI approach impacts these student populations.

### 4.6. Limitations, future directions, and possible modifications

There are a few limitations to our TTT-GI activity design and implementation that we would address in future implementations in the same hyflex Human Anatomy and Physiology course. First, prior to the semester in which this TTT-GI activity was implemented, a lesson dedicated to the gut microbiome was not included in the Human Anatomy and Physiology course that was the focus of this study. Given the overarching learning objectives and other lessons that needed to be covered in the course, a single lesson was all that could be devoted to our novel TTT-GI activity. The results from the 50-min section (Table 3) suggest that spacing this activity over two lessons may have been more beneficial for students. Additionally, because the focus of this TTT-GI activity was not discussed in the textbook used for the course, students were required to read scholarly papers as their pre-reading assignment (Stevens and Hume, 1998; Leshem et al., 2020). Because reading scholarly literature is a skill that is developed over time, many students likely struggled with this pre-reading assignment. How this impacted student learning for the TTT-GI activity is unknown and is something that we would like to explore further. While we provided students with guided reading questions, we may also try to provide additional resources to support their reading of scholarly literature in the context of this lesson or others in the course. These could include dedicated class time where students practice reading and interpreting primary literature and/or the use of online tutorials for students to learn how to read scholarly articles (Gillen et al., 2004; Hoskins et al., 2011; Liao, 2017; Carmichael and Allison, 2019). Finally, because cooperative learning is integral to our TTT-GI activity, we may consider establishing roles for individual members of each group, similar to other group-based pedagogies (Hanson, 2006; Beichner et al., 2007; Gaffney et al., 2008; Ott et al., 2018). These roles are thought to help establish team interdependence (Johnson and Johnson, 1989; Johnson et al., 1991) and may be particularly beneficial by providing structure in a high-enrollment course. For a group of three, we propose the following roles: a team leader, who is responsible for keeping the team on task; a recorder, who is responsible for writing answers on the guided-inquiry document; and a kit manipulator, who is responsible for passing out kit components and/or manipulating aspects of the kit. How specific roles within student teams impact student learning from a TTT-GI activity is an additional area for future investigation.

If this activity were to be implemented in a different course and/or format, additional modifications would likely need to be made. As we found from our analyses, students who had only completed a 100-level introductory biology course did not have as great of learning gains from this activity as students who had completed upper-level biology coursework (Table 4; Supplementary Table 4). Students who have minimal experience with biology coursework may therefore benefit from additional review of foundational topics such as enzyme-substrate interactions, carbohydrate structures, and glycolysis and fermentation pathways, as pre-lesson homework for this activity. Indeed, a review might be beneficial for all students, even those in the Human Anatomy and Physiology course described here, as multiple opportunities for students to engage with specific concepts reinforces their learning (Rovick et al., 1999; Beeber and Biermann, 2007). Physical attributes of the classroom where this TTT-GI activity was implemented may also need to be taken into consideration. While we implemented the activity in a traditional, stadium-seating lecture hall with success, we acknowledge that the classroom was not at maximum capacity given the hyflex nature of the class. This allowed students in attendance to spread throughout the classroom to manipulate the kit. We do not know if the activity would have been as successful if implemented in a fully in-person lecture hall filled to maximum capacity. Regardless, instructors may want to consider seating density when implementing this activity and try to implement in classrooms with tables for students to manipulate the tactile teaching tools. Similarly, the cost of the tactile teaching tools, as well as storage space, are barriers to implementation (Ramirez and Gordy, 2020), especially for a high-enrollment course such as this for which numerous kits are needed. Internal instructional development grants may be helpful to reduce the costs associated with the TTT-GI approach. Given that we observed success in the remote implementation of this TTT-GI activity (Table 3), instructors may also consider implementing the digital version of this activity to alleviate the costs and storage burdens associated with our physical TTT-GI kit.

There were a few limitations to our assessment of this TTT-GI activity. First, student demographic data were all self-reported and we identified numerous individual identities that required us to create bins during our data analysis. This may have skewed our demographic survey results. Similarly, since we tried to provide criteria for our demographic variables, such as context for the different educational levels, it is possible that a true first year student at the institution selected second year or higher because they had completed the necessary credits to put them at that level. The other limitation is that our questions assessing the learning objectives of this activity did not undergo validation. While we saw gains in 8 out of 9 of these questions, it is unclear how unintended aspects of the questions may have impacted student performance. Further, since we used a pre−/post-assessment schema, we assume that many students guessed on the pre-assessment and that any correct answers may not actually reflect students’ knowledge related to the question. Finally, since students saw the same questions on both the pre-and post- assessment, we are unsure of how the testing effect may have factored into our results (Chan et al., 2006; Hinze and Wiley, 2011). We suspect, however, that the testing effect had a minimal impact on our results since there were at least 13 weeks between the pre-and post-assessments.

While were able to collect preliminary data in this experiment that suggest that this TTT-GI activity is potentially beneficial for first-generation and students with one or more disabilities (Table 4; Supplementary Table 3), our subpopulations of diverse students are small and warrant further investigation. Future directions for this project therefore include expansion to additional classes as well as other university campuses with better representation of underrepresented students (e.g., minority serving institutions). Additionally, we may also consider how to adapt this activity for implementation in high school classrooms, given the adoption of new curricular standards in the sciences, namely the Next Generation Science Standards (NGSS). NGSS emphasizes student-centered inquiry. As such, this project could have great potential to promote student learning while simultaneously addressing issues of access and equity for NGSS implementation (Harris et al., 2017). The above future directions directly align with the goals of the TTT-GI approach for improving access and accessibility to novel and interesting active learning opportunities that promote student learning (Ramirez and Gordy, 2020).

## Data availability statement

The raw data supporting the conclusions of this article will be made available by the authors, without undue reservation.

## Ethics statement

The studies involving human participants were reviewed and approved by The University of North Carolina at Chapel Hill Institutional Review Board. The patients/participants provided their written informed consent to participate in this study.

## Author contributions

PS, KF, EM, and LO designed this TTT-GI activity. PS, NC, and LO implemented the activity, and collected and analyzed the data. PS, KF, NC, and LO wrote the first draft of the manuscript. All authors contributed to the article and approved the submitted version.

## Funding

This work was supported by the National Science Foundation under RCN-UBE INCUBATOR #2018668.

## Conflict of interest

The authors declare that the research was conducted in the absence of any commercial or financial relationships that could be construed as a potential conflict of interest.

## Publisher’s note

All claims expressed in this article are solely those of the authors and do not necessarily represent those of their affiliated organizations, or those of the publisher, the editors and the reviewers. Any product that may be evaluated in this article, or claim that may be made by its manufacturer, is not guaranteed or endorsed by the publisher.
