# Supplementary material for: A gut microbiome tactile teaching tool and guided-inquiry activity promotes student learning
Source: Front Microbiol. 2022 Dec 22;13:966289. doi: 10.3389/fmicb.2022.966289 (PMC9813521; doi:10.3389/fmicb.2022.966289)
Supplement: Supplementary file 1 [file Data_Sheet_1.DOCX]

# A Gut Microbiome Tactile Teaching Tool and Guided-Inquiry Activity Promotes Student Learning

**Parker T. Shoaf^1^, Katie S. French^2^, Noah J. Clifford^1^, Erin A. McKenney^3‡^, Laura E. Ott^14‡*^**

^1^Department of Biology, University of North Carolina at Chapel Hill, Chapel Hill, NC, USA

^2^Department of Forestry and Environmental Resources, North Carolina State University, Raleigh, NC, USA

^3^Department of Applied Ecology, North Carolina State University, Raleigh, NC, USA

^4^Carolina Biology Education Research Laboratory, University of North Carolina at Chapel Hill, Chapel Hill, NC, USA

^‡^These authors have contributed equally to this work and share senior authorship

*** Correspondence:**
**Laura E. Ott**
 [leott@email.unc.edu](mailto:leott@email.unc.edu)

Supplementary Material

1. Demographics Questionnaire
2. Supplemental Table 1: Comparison of 50-minute and 75-minute sections
3. Supplemental Table 2: Pre- and post-assessment question performance
4. Supplemental Table 3: Comparison of student learning gains based on demographic variables
5. Supplemental Table 4: Upper-level biology course completion of diverse student populations
6. Representative Assessment Questions

**Demographics Questionnaire**

1. Which best describes you?
   1. I transferred from UNC from a 2-year institution
   2. I transferred from UNC from a 4-year institution
   3. I started my college experience at UNC
   4. I earned college credit from high school (e.g., dual enrollment or AP exam)
2. Which best describes you?
   1. First year student (under 30 credit hours total)
   2. Sophomore (31-60 credits)
   3. Junior (61-90 credits)
   4. Senior (91+ credits)
   5. Other (please specify)
3. What is your major?
   1. Biology (BS, BA, Quantitative)
   2. Biochemistry
   3. Psychology and Neuroscience
   4. Pre-nursing
   5. Pre-nutrition
   6. Pre-medicine
   7. Pre-physician’s assistant
   8. Non-degree seeking student
   9. Other (please specify)
4. What are your career goals?  (50 characters or less)
5. What are your pronouns? (Select all that apply)
   1. She/her
   2. He/him
   3. They/them
   4. Other (please specify)
   5. Prefer not to disclose
6. Check one or more options that reflect your gender:
   1. Man
   2. Woman
   3. Non-binary
   4. Transgender
   5. Intersex
   6. Two Spirit
   7. Gender non-conforming
   8. Other (please specify)
7. Check one or more options that best describes you:
   1. Asian or Pacific Islander
   2. Black or African American
   3. Hispanic or Latino
   4. Native American or Alaskan Native
   5. White or Caucasian
   6. Multiracial or Biracial
   7. Other (please specify)
   8. Prefer not to disclose
8. Are you a first-generation college student?  (i.e., neither of your parents completed a four-year college degree)
   1. Yes
   2. No
   3. Unsure
9. Do you identify as having a disability as defined under the Americans with Disabilities Act (<https://adata.org/faq/what-definition-disability-under-ada>)? (Select all that apply)
   - 1. Yes, physical
     2. Yes, hearing
     3. Yes, mental
     4. Yes, physical
     5. Yes, visual
     6. Yes, self-identify
     7. No
     8. Prefer not to answer
10. Which of the following biology courses have you previously taken? (Select all that apply)
    1. BIOL 101 (at UNC)
    2. BIOL 101 (AP/IB credit)
    3. BIOL 101 (Equivalent Transfer)
    4. BIOL 201
    5. BIOL 202
    6. BIOL 205
    7. BIOL 4XX
11. Which best describes you?
    1. I took BIOL 101 at UNC
    2. I tested out of BIOL 101 (placement test or AP test)
    3. I took a BIOL 101 equivalent at a community college
    4. I took a BIOL 101 equivalent at another 4-year institution
    5. Other (please specify)

**Supplemental Table 1:** Comparison of the 50-minute and 75-minute sections

| **Characteristic** | **50-minute section** | **75-minute section** |
| --- | --- | --- |
| Class meeting pattern | Monday, Wednesday, and Friday, 50 minutes each | Tuesday and Thursday, 75 minutes each |
| Time of class | 11:15 AM-12:05 PM | 12:30-1:45 PM |
| Attendance options | In-person or virtual | In-person or virtual |
| Total enrollment | 183 | 169 |
| Consenting participants | 90 | 92 |
| Number of peer instructors | 9 | 9 |
| Instructor | Ph.D. level instructor (LEO) | Ph.D. level instructor (LEO) |
| Classroom assigned | Lecture hall with traditional stadium seating | Lecture hall with traditional stadium seating |
| Activity implementation | Entirety of a single class session plus review for 10 minutes at the start of next class session | Entirety of single class session |
| Review session | Optional virtual review session facilitated by peer instructor (PTS) that was recorded and posted to the course learning management site. | No formal review session provided, but students could discuss content with peer instructors informally |
| Population of transfer students | 7 (7.78%) | 17 (18.5%) |
| Population of first year students | 5 (5.56%) | 10 (10.9%) |
| Population of upper-level students (sophomore-level or higher) | 85 (94.4%) | 82 (89.1%) |
| Population of female students | 58 (64.4%) | 62 (67.4%) |
| Population of PEER students | 20 (22.2%) | 22 (23.9%) |
| Population of first-generation students | 17 (18.9%) | 23 (25.0%) |
| Population of students with one or more disability | 6 (6.67%) | 5 (5.43%) |
| Population of students who had completed upper-level coursework | 67 (74.4%) | 59 (64.1%) |
| Population of students who had only completed Intro Bio | 23 (25.6%) | 33 (35.9%) |
| Population of Life Science majors | 47 (52.2%) | 45 (48.9%) |
| Population of pre-health majors | 43 (47.8%) | 46 (50.0%) |

**Supplemental Table 2:** Student (n=182) pre-assessment and post-assessment performance on questions that assess the student learning objectives. Scores are represented as percent (%) correct. Data were analyzed using a paired t-test.

| **Question** | **Learning Objective(s)** | **Pre-assessment score (mean±SD)** | **Post-assessment score (mean±SD)** | **p-value** |
| --- | --- | --- | --- | --- |
| 1 | LO1 | 46.70±50.03 | 89.01±31.36 | <0.0001 |
| 2 | LO3 | 71.98±45.03 | 85.71±35.09 | 0.0017 |
| 3 | LO3 | 32.97±47.14 | 78.57±41.15 | <0.0001 |
| 4 | LO2 | 17.58±38.17 | 58.79±49.36 | <0.0001 |
| 5 | LO1; LO3 | 38.46±48.78 | 91.76±27.58 | <0.0001 |
| 6 | LO3 | 14.29±35.09 | 75.27±43.26 | <0.0001 |
| 7 | LO3 | 45.60±49.94 | 35.71±48.05 | 0.0548 |
| 8 | LO1 | 54.40±49.94 | 89.56±30.66 | <0.0001 |
| 9 | LO3 | 31.32±46.51 | 59.89±49.15 | <0.0001 |

**Supplemental Table 3:** Comparison of student learning gains based on demographic variables. Learning gains represent the average pre-assessment score (%) subtracted from the post-assessment score (%) for all nine assessment questions. Data is represented as mean (±SD)

| **Demographic variables** | | **Gains (%)** | **N** |
| --- | --- | --- | --- |
| ***Transfer status*** |  |  |  |
|  | Transfer student (from 2-year or 4-year institution) | 27.31±26.41 | 24 |
|  | Direct entry student | 35.65±24.51 | 158 |
| ***Educational level*** |  |  |  |
|  | First year students (0-30 credits) | 18.52±31.89 | 15 |
|  | Second year students (31-60 credits) | 34.97±23.76 | 95 |
|  | Third year students (61-90 credits) | 36.27±23.18 | 53 |
|  | Fourth year students (91+ credits) | 46.67±22.30 | 15 |
| ***Gender identity*** |  |  |  |
|  | Female | 32.96±24.36 | 120 |
|  | Male | 37.29±26.24 | 59 |
|  | Non-binary | 44.44±11.11 | 3 |
| ***Racial/ethnic identity*** |  |  |  |
|  | PEER^[[1]](#footnote-2)^ students | 29.89±24.96 | 42 |
|  | Non-PEER students | 36.29±24.50 | 139 |
| ***First generation status*** |  |  |  |
|  | First generation student | 36.94±22.70 | 40 |
|  | Non-first generation student | 33.73±25.58 | 140 |
| ***Disability status*** |  |  |  |
|  | Students with one or more disability | 41.41±23.88 | 11 |
|  | Students with no reported disability | 34.63±24.92 | 163 |
| ***Pre-requisite coursework*** |  |  |  |
|  | Students who completed upper-level Biology coursework | 37.92±23.06 | 126 |
|  | Students who only completed Intro Biology | 26.98±27.20 | 56 |
| ***Degree plan*** |  |  |  |
|  | Life science majors – biochemistry (B.S.), biology (B.A., B.S.) and psychology and neuroscience (B.S.) | 35.27±24.69 | 92 |
|  | Pre-health majors – pre-nursing, pre-dentistry, pre-med, pre-nutrition, pre0physician’s assistant | 33.83±25.29 | 89 |

**Supplemental Table 4:** Upper-level biology course completion rates by diverse student populations in our sample. Upper-level biology courses are defined as a 200-level or above course.

| **Demographic Variable** | | **% with Upper-Level Biology Coursework** |
| --- | --- | --- |
| ***Transfer status*** |  |  |
|  | 2-/4-year transfer students | 58.33% |
|  | Direct entry students | 70.89% |
| ***Educational level*** |  |  |
|  | First year students (0-30 credits) | 6.67% |
|  | Second year students (31-60 credits) | 72.63% |
|  | Third year students (61-90 credits) | 77.36% |
|  | Fourth year students (91+ credits) | 86.67% |
| ***Racial/ethnic identity*** |  |  |
|  | PEER^1^ | 35.71% |
|  | non-PEER | 79.14% |
| ***Gender identity*** |  |  |
|  | Men | 77.59% |
|  | Women | 65.00% |
|  | Non-binary | 66.67% |
| ***First-generation status*** |  |  |
|  | First generation | 55.00% |
|  | Non-first generation | 74.29% |
| ***Disability status*** |  |  |
|  | One or more disability | 72.71% |
|  | No disabilities | 69.94% |
| ***Degree plan*** |  |  |
|  | Life science majors – biochemistry (B.S.), biology (B.A., B.S.) and psychology and neuroscience (B.S.) | 85.87% |
|  | Pre-health majors – pre-nursing, pre-dentistry, pre-med, pre-nutrition, pre0physician’s assistant | 50.56% |
| ***Mode of attendance*** |  |  |
|  | In-person | 68.75% |
|  | Remote | 65.00% |
|  | Unsure | 80.77% |
| ***Class section enrolled*** |  |  |
|  | 75-minute class | 64.13% |
|  | 50-minute class | 74.44% |

**Supplemental Materials 6:** Representative Assessment Questions

Morgan is considered clinically obese and recently had their annual physical. During their appointment, Morgan’s physician suggested that they have an intestinal metagenomics evaluation performed to determine the composition of their gut microbial community.

**[SLO-3] Which of the following best explains how their gut microbiota may be contributing to Morgan’s obesity?**

- 1. Morgan’s gut microbiota is enriched with bacteria that ferment carbohydrates to produce acetate.
  2. Morgan’s gut microbiota is enriched with bacteria that ferment carbohydrates to produce propionate.
  3. Morgan’s gut microbiota is enriched with bacteria that ferment carbohydrates to produce butyrate.

**[SLO-3] In an attempt to change their diet, Morgan starts to consume more cellulose-rich foods. To gain the benefit of this dietary change, they need to ensure that they have microbes in their gut microbiota that express enzymes capable of breaking:**

- 1. Alpha 1,4 bonds
  2. Alpha 1,6 bonds
  3. Beta 1,4 bonds
  4. Two or more of the above
  5. None of the above

**[SLO-2] After two weeks of regularly consuming more cellulose in their diet, Morgan should expect to find a more robust population of _________ in their gut microbiota.**

- 1. Actinobacteria
  2. Bacteriodetes
  3. Gammaproteobacteria
  4. Firmicutes

**[SLO-1,3] Because of Morgan’s age (47), their physician also recommends that they schedule their first routine colonoscopy. The procedure reveals numerous pre-cancerous polyps. If you were Morgan’s gastroenterologist, what would you recommend?**

- 1. Eat foods that favor the production of acetate
  2. Eat foods that favor the production of butyrate
  3. Eat foods that favor the production of propionate
  4. Eat foods that favor the production of acetate and propionate
  5. Eat foods that favor the production of acetate and butyrate
  6. Eat foods that favor the production of propionate and butyrate

**[SLO-1]** ***Clostridium difficile* (*C. diff*) is a bacterium capable of interrupting the normal healthy bacteria in the digestive tract, particularly the colon. A common treatment for those with this infection is a fecal transplant where the correct bacteria are reintroduced. What would be the effect on an individual who consumes a vegetable-based diet if they were given a fecal transplant from someone who primarily eats meat?**

- 1. The patient would initially have difficulty processing plant-based products and producing the necessary fermentation products.
  2. The patient would experience reduced inflammation due to the increased abundance of microorganisms responsible for the production of SCFA.
  3. The patient would not have any physiological changes as a result of their diet changes

1. Asai (2020). *Cell*, 181(4), 754-757 [↑](#footnote-ref-2)
